# Analyzing time‐ordered event data with missed observations

**DOI:** 10.1002/ece3.3281

**Published:** 2017-08-09

**Authors:** Adriaan M. Dokter, E. Emiel van Loon, Wimke Fokkema, Thomas K. Lameris, Bart A. Nolet, Henk P. van der Jeugd

**Affiliations:** ^1^ Dutch Centre for Avian Migration and Demography Netherlands Institute of Ecology Wageningen The Netherlands; ^2^ Department of Animal Ecology Netherlands Institute of Ecology Wageningen The Netherlands; ^3^ Theoretical and Computational Ecology University of Amsterdam Amsterdam The Netherlands; ^4^ Conservation Ecology University of Groningen Groningen The Netherlands

**Keywords:** fecal output, interval time series, missing data, mixture model, observation protocol, probability of detection

## Abstract

A common problem with observational datasets is that not all events of interest may be detected. For example, observing animals in the wild can difficult when animals move, hide, or cannot be closely approached. We consider time series of events recorded in conditions where events are occasionally missed by observers or observational devices. These time series are not restricted to behavioral protocols, but can be any cyclic or recurring process where discrete outcomes are observed. Undetected events cause biased inferences on the process of interest, and statistical analyses are needed that can identify and correct the compromised detection processes. Missed observations in time series lead to observed time intervals between events at multiples of the true inter‐event time, which conveys information on their detection probability. We derive the theoretical probability density function for observed intervals between events that includes a probability of missed detection. Methodology and software tools are provided for analysis of event data with potential observation bias and its removal. The methodology was applied to simulation data and a case study of defecation rate estimation in geese, which is commonly used to estimate their digestive throughput and energetic uptake, or to calculate goose usage of a feeding site from dropping density. Simulations indicate that at a moderate chance to miss arrival events (*p* = 0.3), uncorrected arrival intervals were biased upward by up to a factor 3, while parameter values corrected for missed observations were within 1% of their true simulated value. A field case study shows that not accounting for missed observations leads to substantial underestimates of the true defecation rate in geese, and spurious rate differences between sites, which are introduced by differences in observational conditions. These results show that the derived methodology can be used to effectively remove observational biases in time‐ordered event data.

## 1 INTRODUCTION

A common problem with observational data is that records may be incomplete and conditional on the observation process. For example, count data underlying population size estimates (Buckland et al., 2001; Royle, 2004), animal distributions (Fink et al., 2010), or extinction records (Solow, 2005) may be sparse and unevenly distributed in space and time, requiring statistical analyses that correct for observer effort. Incomplete data may also result from compromised detections during the observation process itself (Elphick, 2008). Auditory detections may depend on the level of ambient noise (Simons, Alldredge, Pollock, & Wettroth, 2007) or observer skill (Kendall, Peterjohn, & Sauer, 1996), and visual detections may be compromised because organisms move out of view, hide, or avoid the observer, causing events of interest to remain occasionally unobserved without the observer realizing. Similarly, technology to record event data like accelerometers on GPS tags may not be continuously operational (Shamoun‐Baranes et al., 2012), for example, for considerations of energy or memory consumption, leading to missing observations during down‐time. Correction for missingness in data due to a compromised detection process is the topic of this study.

Observations in ecology often consist of simple counts of events and their timing (Altmann, 1974). Estimating time‐to‐event in a time series with missing values may be relevant in any process with observations of recurring discrete events. This type of data is common in behavioral protocols collected by field observers or devices, such as observations of diving intervals in birds or mammals (Nolet, Wansink, & Kruuk, 1993; Wilson, Pütz, Charrassin, & Lage, 1995), recordings of vigilance bouts or scanning frequency in socially foraging animals (Hirschler, Gedert, Majors, Townsend, & Hoogland, 2016), observations of animal defecation events (Owen, 1971; Ydenberg & Prins, 1981), nest visit rates (Lendvai et al., 2015), or data on prey captures. Estimating time‐to‐event data with missing observations plays a role not only in behavioral protocols, but in any cyclic or recurring process where discrete outcomes are observed, such as peaks in environmental or animal cycles (Sinclair et al., 1993), tree‐ring observations (Bradley, 2011), or cyclic sedimentation (Brandon, Woodruff, Donnelly, & Sullivan, 2014).

In this study, we focus on count data that is ordered in time series, as typically the case in behavioral protocols. We will derive how repeated event observations on individuals may be used to correct for missed detections, using a statistical framework based on a newly derived probability density function (pdf) of observed intervals that contains explicit components to account for intervals containing a missed observation. The method is made available in the R‐package intRvals. We will demonstrate the utility of the method and the package in a case study that deals with estimating defecation rates of animals in the wild, as well in a simulation study. While the authors have used the method to deal with defecation rate estimation in geese, the framework is general to any observational process that aims to estimate and compare the mean and variance of occurrence rates in time series of distinct events while correcting for missed detections, either by devices or human observers.

In wildlife studies, defecation rate estimates are widely used to assess their food harvest rate, food assimilation, and energy budgeting across different habitats and diets (Besiktepe & Dam, 2002), or to calculate usage of feeding sites from dropping density (Owen, 1971). As bites and bite sizes are difficult to observe directly, fecal output is a much more accurate measure of food intake, which can be calculated as the product of average dropping mass and defecation rate. In the case of grazing waterfowl, a widely used field protocol for assessing the defecation rate has been the “direct interval method,” in which arrival times of successive defecations are recorded in the same individual (Owen, 1971; Ydenberg & Prins, 1981); however, this method does not account for the possibility of missed observations. We will highlight in a case study how our method can be an improvement when observational conditions are challenging and the probability of missed defecation observations cannot be assumed to be zero.

### 1.1 Probability density function of an observed interval distribution

**Figure 1:**
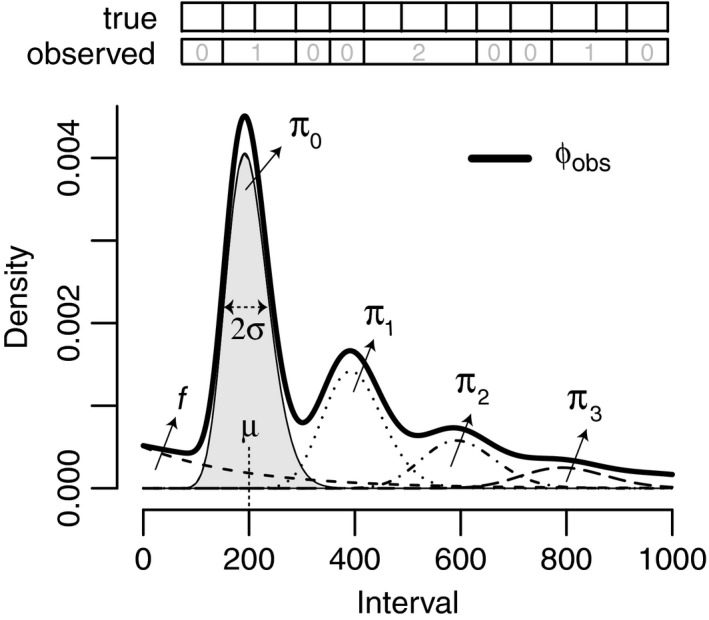
Example probability density function $\varphi_{\mathrm{obs}}\left(x|\mu \;=\;200,\;\sigma \;=\;40,\;p\;=\;0.5,\;f\;=\;0.2\right)$ for an observed interval distribution and its components. The area under each curve (shaded in gray for the fundamental component) equals (1 − f)*π_i_, with π_i_ given by equation (2), and f for the (optional) exponential Poisson process component. The length of the bars at the top of the figure indicate the true and observed interval lengths, where the gray number in the bars indicates the number of consecutively missed arrivals i

We define an arrival as a distinct event that can be observed or recorded on an organism, which may be a behavior, activity, or physical state that is short in duration, therefore to which a distinct time stamp can be assigned. Observers (or devices) are assumed to record arrival times, and from those inter‐arrival times or intervals can be calculated, as illustrated in Figure 1. We further assume that arrivals occur at a certain average stationary rate, equaling the average number of arrivals per unit of time. Our goal is to estimate the mean inter‐arrival time and its standard deviation, in situations where a certain proportion of arrivals will not be observed. In the case that arrivals are fully stochastically independent, we are dealing with a Poisson process, for which the inter‐arrival times are exponentially distributed (Tijms, 2003). In many cases, however, events occur at a specific rate, such that the time between arrivals varies around a mean μ, as in *x*_*t*_ = μ + ε_*t*_ with ε_*t*_ white noise with a certain fixed variance. In this case, the arrival process is no longer a Poisson but rather an autoregressive process or AR(0) process, where the inter‐arrival times are assumed to follow a normal distribution (Manly, 2009).

A real‐life example of such a process is defecation rates by animals. After a defecation, it takes time before new feces accumulates in the rectum, after which it will defecate again. As a result, the arrivals of a subsequent defecations are not random in time but interrelated, with the inter‐arrival time depending on the rate of fecal throughput in the intestines.

### 1.2 Interval data and the gamma distribution

A useful distribution to model inter‐arrival times, *x*, is the Gamma distribution with probability density function (pdf) $\varphi_{\mathrm{true}}^{\Gamma }\left(x|\mu ,\sigma \right)$, given by:

(1) $$\begin{array}{cc} & \text{Gamma}\left(x|a,s\right)\;=\;\frac{1}{\Gamma \left(a\right)}s^{-a}x^{a-1}e^{-\frac{x}{s}}\\ & \varphi_{\mathrm{true}}^{\Gamma }\left(x|\mu ,\;\sigma \right)∼\text{Gamma}\left(x|\mu^{2}/\sigma^{2},\;\sigma^{2}/\mu \right) \end{array}$$

with μ the mean, σ the standard deviation, Γ the gamma function, and *x* the time interval between subsequent arrivals. When μ ≫ σ the limiting distribution of the gamma distribution is the standard normal distribution with pdf $\varphi_{\mathrm{true}}^{N}\left(x|\mu ,\;\sigma \right)∼N\left(\mu ,\;\sigma^{2}\right)$, and when μ ≈ σ the limiting distribution is the exponential distribution with pdf $\varphi_{\mathrm{true}}^{e}\left(x|\mu \right)\;=\;\varphi_{\mathrm{true}}^{\Gamma }\left(x|\mu ,\mu \right)\;=\;e^{-x/\mu }/\mu$. This property makes the Gamma distribution suited to model a wide range of arrival processes: It spans the range of random Poisson's processes (the limit of exponentially distributed arrival intervals) to autoregressive processes (the limit of normally distributed arrival intervals).

### 1.3 A probability density function for observed intervals

The theoretical probability density function φ_obs_ of *observed* arrival intervals in a scenario where the chance to miss an arrival is nonzero, will be a superposition of multiple components referring to different sets of observed intervals, separated by the number of missed arrivals they contain, as illustrated in Figure 1. The component for the interval set containing no missed arrivals will have a total cumulative probability of (1 − *p*), with *p* the probability to miss an arrival. Of the proportion *p* of true arrivals missed once, again in fraction *p* of the cases a subsequent arrival will be missed for a second time, that is, in a fraction p*p of the cases. The set containing one missed arrival will therefore have a total cumulative probability of (*p* − *p* ^2^). In general, the component π_*i*_ of the pdf referring to interval sets with *i* missed consecutive arrivals thus equals

(2) $$\pi_{i}\;=\;p^{i}-p^{i\;+\;1}$$

One may easily verify that the sum of the cumulative density of all components of the observed pdf $\sum_{i=0}^{\infty }\pi_{i}=1$ for any value *p* between 0 and 1, as required for a pdf.

The expected value of the interval set without missing intervals, $E\left(\pi_{0}\right)$, equals μ. From this follows that for the sets with missing intervals $E\left(\pi_{i}\right)$ = $\left(i\;+1\right)\mu$. The width of component *i* will be broadened relative to the fundamental, as it results from the addition of (*i* + 1) intervals, each with associated standard deviation. The width of component *i* can thus be calculated by standard uncertainty propagation in the case of addition, such that we may write for the observed probability density function φ_obs_

(3) $$\begin{array}{cc} \varphi_{\mathrm{obs}}\left(x|\mu ,\;\sigma ,\;p\right) & \;=\;\sum_{i\;=\;0}^{\infty }\varphi_{\mathrm{obs}}\left(x,\;i|\mu ,\;\sigma ,\;p\right)\\ \varphi_{\mathrm{obs}}\left(x,\;i|\mu ,\;\sigma ,\;p\right) & \;=\;\pi_{i}\;\varphi_{\mathrm{true}}^{\Gamma }\left(x|\left(i\;+1\right)\mu ,\;\sqrt{i\;+1}\sigma \right) \end{array}$$

Equation (3) is mathematically analogous to a mixture model (Bishop, 2006), with mixture components $\varphi_{\mathrm{true}}^{\Gamma }$ and mixing coefficients π_*i*_. Conventional mixture models aim to estimate the means, variances, and mixture coefficients for typically an unknown but finite number of components. Equation (3) differs primarily from the conventional mixture model by the constraining relations that exist between the means, variances, and magnitudes of the mixing coefficients of each the components, as well by the infinite instead of finite number of components.

We can add to the pdf of equation (3) a distinction between within‐subject and total variation (σ_*w*_, σ, respectively), which is meaningful in a limit where σ_*w*_ **< σ. This inequality also implies σ_*w*_ < μ as the lower limit for σ is given by random processes in which μ ≈ σ. When within‐subject variation is distinguishable, the pdf $\varphi_{\mathrm{obs}}\left(x|\mu ,\;\sigma_{\mathrm{within}},\;p\right)$ will tend to an autoregressive process, that is, a sum of normal distributed terms, which is easily convoluted with a normal distribution to account for between‐subject variation. The pdf for all subjects combined thus becomes

(4) $$\varphi_{\mathrm{obs}}\left(x|\mu ,\;\sigma ,\;\sigma_{w},\;p\right)=\sum_{i\;=\;0}^{\infty }\pi_{i}\;\varphi_{\mathrm{true}}^{\Gamma }\left(x|\left(i\;+1\right)\mu ,\sqrt{i\sigma_{w}^{2}\;+\;\sigma^{2}}\right)$$

with $\sigma =\sqrt{\sigma_{w}^{2}\;+\;\sigma_{b}^{2}}$. One may verify that when σ = σ_*w*_ (σ_*b*_ = 0), equation (3) is recovered as required.

### 1.4 Accounting for stochastic background processes

It is conceivable that, superimposed on an autoregressive process, a fraction of events is triggered by an additional stochastic process. We will refer again to animal defecation for an example. Herbivorous waterfowl like geese are known to defecate at an interval related to their rate of digestive throughput, and typically have defecation intervals with μ > σ (Bédard & Gauthier, 1986; Prop, Van Marken Lichtenbelt, Beekman, & Faber, 2005). Nonetheless, random events can trigger defecations, for example, disturbances, take‐off into flight, or aggressive interactions, which tend to be accompanied with defecation events (own unpublished observations by the authors). Generally, steady‐state behaviors or activities of animals always have a chance of being interrupted, for example, by interactions with competitors, predators, or by fluctuations in their abiotic environment, which makes it important to account for such randomness.

When these background stochastic arrivals are random in time (i.e., a Poisson process), they will produce an exponential interval distribution. For this subset of random arrivals, short intervals near zero are the most common by nature of the exponential distribution (Tijms, 2003). However, for a main arrival process having μ > σ, the probability of short observed intervals is very low in eq. (3), and any observed interval near zero will effectively behave as a major outlier to the theoretical distribution. In these cases, it is important to accommodate these outliers in a pdf that describes a mixture process that includes a small fraction *f* of random arrivals (with an observed exponential distribution $\varphi_{\mathrm{obs}}\left(x|\mu ,\mu ,p\right)$; note that φ_obs_ represents a gamma distribution which simplifies to an exponential distribution if μ = σ):

(5) $$\varphi_{\mathrm{obs}}\left(x|\mu ,\;\sigma ,\;p,\;f\right)\;=\;\left(1\;-\;f\right)\cdot \varphi_{\mathrm{obs}}\left(x|\mu ,\;\sigma ,\;p\right)\;+\;f\cdot \varphi_{\mathrm{obs}}\left(x|\mu ,\;\mu ,\;p\right)$$

As a boundary condition, we will assume that the random background component has the same mean interval length as the main component, such that the mean interval length remains identical to equation (3). The biological interpretation of this assumption is that the infrequent stochastic interruptions of the animal's behavior of interest do not change the long‐term average rate of the behavior maintained by the animal.

## 2 METHODS

### 2.1 Maximum‐likelihood estimation and model comparison

For a set of {*x*_*j*_} observed intervals, we calculated a log‐likelihood L

(6) $$L\left(\mu ,\;\sigma ,\;p,\;f|\left\{x_{j}\right\}\right)=\sum_{j}\log\varphi_{\mathrm{obs}}\left(x_{j}|\mu ,\;\sigma ,\;p,\;f\right)$$

which can be maximized with respect (μ*,* σ, *p*,* f*) using standard numerical procedures, producing an estimate of these parameters. Mixture models are usually estimated by the iterative expectation maximization (EM) algorithm (Bishop, 2006; Dempster, Laird, & Rubin, 1977), in which the mixture coefficients are considered unobserved latent variables that are estimated in a step (expectation‐ or E‐step) that is separate from the estimation of the main model parameters (maximization‐ or M‐step). Our model is essentially a mixture model with additional constraints on the relative values of the mixture coefficients and their means and variances. To the authors knowledge, current statistical packages for estimating mixture models (e.g., mixtools (Benaglia, Chauveau, Hunter, & Young, 2009) or FlexMix (Leisch, 2004)) do not allow specification of such constraints on both the mixture coefficients and model parameters, a restriction that is lifted in our new package intRvals (but see normalmixMMIc in R‐package mixtools for specification of linear constraints on means and variances in normal mixture models, (Benaglia et al., 2009)). The EM algorithm is particularly efficient for normal mixtures, as in this case, analytical solutions exist for the M‐step. Maximum‐likelihood estimators for the Gamma distribution, however, do not have a closed form (Choi & Wette, 1969), and its estimation already relies on gradient‐based methods. Because our pdf contains Gamma distributions by default, we did not implement an EM algorithm for maximizing equation (6) and use gradient‐based methods directly instead. Because model parameters *f* and *p* are constrained between 0 and 1, we introduced a binomial link function in the maximum‐likelihood estimator to constrain their value to this domain. We found the estimator to converge reliably and quickly, likely because the additional constraints greatly reduce the number of free parameters relative to conventional mixture models.

To compare the means and variance of two populations, we can use the optimized mean *m* and standard deviation *s* in a standard Student's *t* test and *F* test, respectively. Although the model parameters are estimated using Gamma distributions and the tests are derived under assumptions of normality, both tests are known to be still robust (Grice & Bain, 1980). Only when sample sizes are very small and the gamma distribution's shape parameter is small (μ ~ σ), the tests may be less appropriate (Shiue, Bain, & Engelhardt, 1988; Tripathi, Gupta, & Pair, 1993). When a random background Poisson process fraction *f* is included to allow for interval lengths near zero, we reduced the effective degrees of freedom in the tests by the same factor *f*. To compare model fits on the same population, we use a likelihood ratio test based on calculated deviance values. As our candidate models are nested, we may apply Wilks’ theorem (Wilks, 1938) and assume the deviance has an approximate χ^*2*^ distribution with degrees of freedom Δ*n*_param_ (the difference in the number of optimized free parameters between two models). In practice, pdf values will be numerically identical when the infinite sum in equation (6) is capped at a finite integer, that is, in our numerical case studies, we ran the sum up to *i* = 5. The pdf was truncated and renormalized at the approximately mean observation bout length, equal to 15 min.

We note that package intRvals contains deviance tests for comparing different competing interval models that may be fitted on the same interval data set, for example, to compare the assumption of gamma‐distributed intervals versus normal distributed intervals (i.e., replacing $\varphi_{\mathrm{true}}^{\Gamma }$ with $\varphi_{\mathrm{true}}^{N}$ in eq. (4)), or to test the validity of the capping of the sum in equation (6).

### 2.2 Partitioning within‐ and between‐subject variation

Given a model fit, by equation (3), we have the decomposition of the likelihood of an interval observation into partial likelihoods $\varphi_{\mathrm{obs}}\left(x,\;i|\mu ,\;\sigma ,\;p\right)$, that is, each of the components illustrated in Figure 1. If the amplitude of partial likelihood $\varphi_{\mathrm{obs}}\left(x,\;i\;=\;0|\mu ,\;\sigma ,\;p\right)$ is at least a proportion 0.9 of the sum of all terms $\sum_{i}\varphi_{\mathrm{obs}}\left(x,\;i|\mu ,\;\sigma ,\;p\right)$, an interval x is considered to be fundamental (not containing a missed event observation). In other words, fundamental intervals were selected at a 0.9 confidence level. Within‐ and between‐subject variation was estimated on the subset of fundamental intervals only. The value of 0.9 was chosen as compromise between selecting fundamental intervals only, but also retaining sufficient intervals as fundamental for a post hoc analysis. There is ambiguity in its precise choice, and we note that it is good practice to not have inferences rely critically on its value. We fit equation (4) with an initial guess for σ_*w*_, select the subset of fundamental intervals, and update the value σ_*w*_ by estimating it on this subset. Equation (4) is fitted with iteratively updated σ_*w*_ until reaching convergence. We calculate $\sigma_{w}\;=\;s_{w}n_{\mathrm{ind}}/\left(n_{\mathrm{ind}}\;+\;1\right)$ with *s*_*w*_ the uncorrected sample standard deviation of within‐subject centered values (obtained from subtracting the subject's mean value from each observation value), and $n_{\mathrm{ind}}/\left(n_{\mathrm{ind}}+1\right)$ Bessel's correction (Heinz, 2011) with *n*_ind_ the average number of repeated measures per subject. Significance of within‐subject variation was determined by testing for a random effect of subject against a constant null model (van de Pol & Wright, 2009), using the R‐package lme4 (Bates, Mächler, Bolker, & Walker, 2015).

All methodology has been integrated into R‐package “intRvals,” to be downloaded from CRAN (cran.r‐project.org) or Github (github.com/adokter/intRvals). This package can be used to test for the presence of observer effects, fit interval distribution models of both the Gamma and Normal family, convert interval parameters into rate parameters, and test for differences in means and variances in arrival intervals.

## 3 RESULTS

### 3.1 Simulation

We simulated observed intervals for a case with only within‐subject variation (σ_*w*_ = σ), one case with primarily between‐subject variation (σ_*w*_ = 4σ*/5*) and one case with primarily within‐subject variation (σ_*w*_ = σ*/5*), when there is a moderate chance to miss arrival events (*p* = 0.3). Simulated and retrieved parameters are given in Table 1. Retrieved parameter values that were corrected for missed observations are within 1% of their true simulated value, whereas uncorrected means and standard deviations are much higher than the simulated values. These uncorrected parameter estimates were biased upward by a factor 1.3–3 by the intervals containing one or more missed arrivals.

**Table 1:** Parameter retrieval for simulated interval data. In each simulation run, 100 interval observations were generated and parameters retrieved using equations (3) and (4)

param	μ	σ	σ_within_	*p*
**Simulated**	**250**	**50**	**–**	**0.3**
Retrieved (eq. (3))	250 (6)	49 (4)	–	0.30 (0.05)***
Uncorrected	336 (16)	158 (14)	–	–
**Simulated**	**250**	**50**	**10**	**0.3**
Retrieved (eq. (4))	256 (11)	56 (7)	13 (4)**	0.23 (0.04)***
Uncorrected	333 (17)	156 (15)	140 (16)	–
**Simulated**	**250**	**50**	**40**	**0.3**
Retrieved (eq. (4))	252 (8)	52 (7)	36 (4)	0.25 (0.04)***
Uncorrected	336 (16)	159 (14)	148 (15)	–

Means and standard deviations in brackets are given for retrieved parameters over 1000 runs. Significance for including a probability for missed detections (parameter p) and for separating within‐group variance (σ_within_) was tested with a likelihood ratio test against a null model without these terms (*p* values denoted by stars: *<.05, **<.01, ***<.001).

### 3.2 Case study: estimating defecation rates in geese

**Figure 2:**
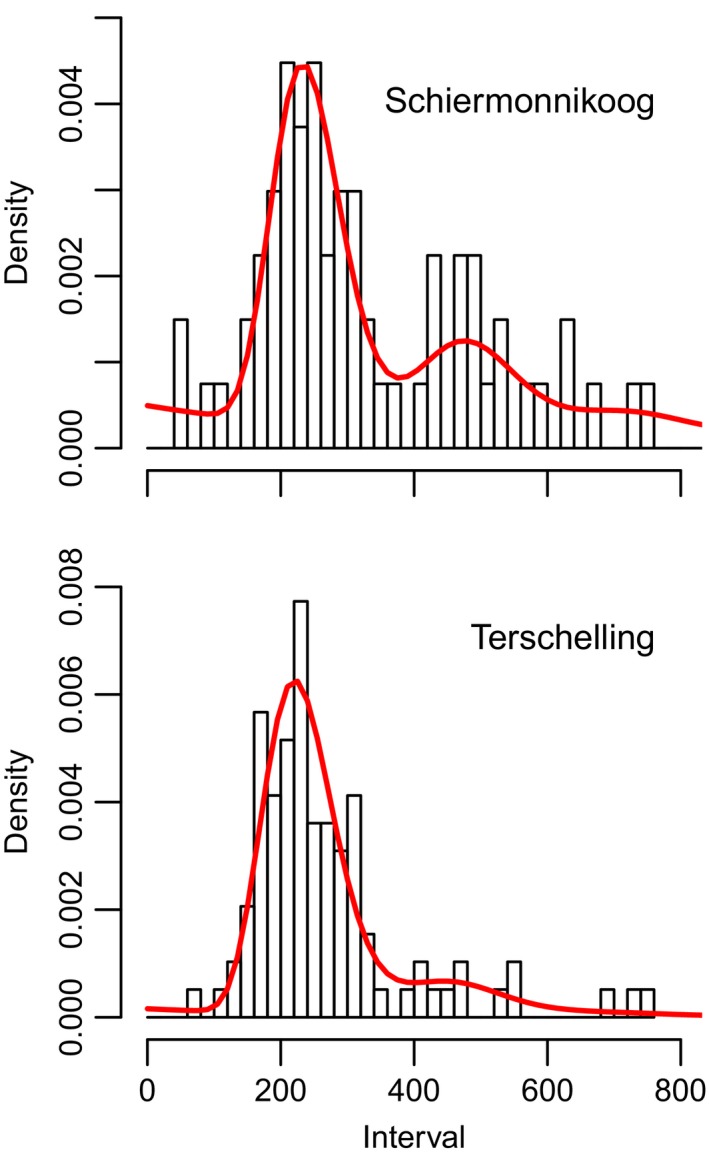
Observed defecation intervals for Brent Geese in May at two sites collected in a 2‐week period, Schiermonnikoog (top, saltmarsh site) and Terschelling (bottom, pasture site). The solid curve is a fit of $\varphi_{\mathrm{obs}}\left(x|\mu ,\;\sigma ,\;p,\;f\right)$ (see eq. (4)) to the interval data. The probability to observe a defecation (1* − p*) is higher at the pasture site

Figure 2 shows distributions of recorded defecation intervals for dark‐bellied brent geese (*Branta bernicla bernicla*) at two different sites, a natural saltmarsh (top, site 1) and agricultural grassland (bottom, site 2). Observers also recorded the time when they had no clear view on the birds’ abdomen, which was a fraction of 0.15 of the total time for pasture versus 0.33 for saltmarsh, reflecting the difficult observational conditions at the saltmarsh.

We fitted different interval models, as summarized in Table 2. Models including a nonzero missed event probability *p* outperformed models that did not, and also inclusion of a random Poisson process background (parameter *f*) was significant on the saltmarsh site. The best models were used for subsequent comparisons of interval length and standard deviation between sites, as summarized in Table 3. The sample mean and standard deviation of the uncorrected observed intervals were much larger than the modeled interval mean and standard deviation. Like in the simulations, their values were biased upwards by intervals containing one or more missed arrivals. The inferred missed event probability was higher at the saltmarsh site than at the pasture site (*p* = 0.27 vs. 0.14). These values are very close to the fraction of observation time that the bird's abdomen was out of view (0.33 vs. 0.15).

**Table 2:** Comparison of interval models within sites

Site	Model	Loglik	*n*_param_	ΔAIC	Sign.
1	$\varphi_{\mathrm{obs}}\left(x|\mu ,\;\sigma ,\;p,\;f\right)$	−430	4	0	
1	$\varphi_{\mathrm{obs}}\left(x|\mu ,\;\sigma ,\;0,\;0\right)$	−437	2	9	***
1	$\varphi_{\mathrm{obs}}\left(x|\mu ,\;\sigma ,\;p,\;0\right)$	−436	3	10	***
1	$\varphi_{\mathrm{obs}}\left(x|\mu ,\;\sigma ,\;0,\;f\right)$	−436	3	11	***
2	$\varphi_{\mathrm{obs}}\left(x|\mu ,\;\sigma ,\;\;p,\;\;f\right)$	−574	4	0	
2	$\varphi_{\mathrm{obs}}\left(x|\mu ,\;\sigma ,\;p,\;0\right)$	−576	3	1	NS
2	$\varphi_{\mathrm{obs}}\left(x|\mu ,\;\sigma ,\;0,\;f\right)$	−583	3	16	***
2	$\varphi_{\mathrm{obs}}\left(x|\mu ,\;\sigma ,\;0,\;0\right)$	−586	2	20	***

Models including both a missed event probability *p* and a random Poisson fraction *f* give the best fit (although inclusion of *f* was only significant at site 1). The best model for each site was used for subsequent between site comparisons. Deviance tests are against the best model for each site (*p* values denoted by stars: *<.05, **<.01, ***<.001).

**Table 3:** Comparison of interval mean and standard deviation between sites (saltmarsh, *n* = 67, pasture *n* = 97 intervals)

Param	Retrieved			Uncorrected		
	Saltmarsh	Pasture	Diff	Saltmarsh	Pasture	Diff
μ	245	233	NS	341	269	***
σ	53	54	NS	186	123	***
σ_within_	24	42	NS	176	113	***
*p*	0.27	0.14	–	–	–	–
*f*	0.20	0.05	–	–	–	–

Not accounting for a nonzero missed event probability p leads to underestimates of the mean, overestimates of the variance, and spurious significant differences in means and variances between sites (*p* values for site comparison denoted by stars: *<.05, **<.01, ***<.001, NS > .05).

## 4 DISCUSSION

We have shown that interval distribution models that account for missed arrival observations can be used to remove biases in the means and variances of interval lengths (and their inverse, the means, and variances in event rates). Simulation results show that the models are robust in retrieving mean, between‐ and within‐subject variances. Observer effects were adequately identified and corrected for under a moderate missed event probability *p* = 0.3, which shows that reliable event rates can be obtained even when observational conditions are challenging, or when data collection is interrupted.

In the defecation case study, the observational conditions were very different at the two sites. Not accounting for missed observations lead to spurious significant differences in means and variances between sites, related to differences in missed event probability *p*. The surface of saltmarshes showed considerable altitudinal variability, and the geese were weary and difficult to approach. Observational conditions were much better at the agricultural grassland site, where geese are accustomed to the presence of humans and can be easily approached and observed without obstructions from a high protective dike surrounding the island. The probability to miss defecation events *p* on the saltmarsh was therefore higher than on pasture.

The retrieved observation probabilities for the sites closely matched the time that abdomens were fully out of view, suggesting an obstructed view was the main reason for missed dropping observations. Accounting for missed observations improved absolute estimates of arrival rates, and prevented spurious results related to difference in observational circumstances. We found that the defecation intervals at the saltmarsh site only appeared longer due to missed defecation events. After correction, the differences between sites became much smaller and were no longer significant for the time periods considered here. Observation time was also used more efficiently, because intervals with missed observations still contribute to the parameter estimates in the model. The analysis further provides an unambiguous way of assigning intervals to the fundamental interval, and folding intervals back to their most likely fundamental interval when they contain a missed observation, functionality which is provided in the intRvals package.

Other methods for estimating interval rates than the “direct interval method” have been proposed to estimate defecation rates. Hourly block counts of observed dropping excretions have been used, in which observation bouts of multiple individuals are used (Bédard & Gauthier, 1986), noting only the number of observed arrivals. The half‐time interval method (Owen, 1971) aims to use observation time more efficiently, by only recording the time until the first defecation α per observation bout on an individual. Both methods do not record exact inter‐arrival times, which are critical to our method; therefore, correction for missed arrivals is not possible when using these protocols. We therefore advise against using these protocols in the field if there is a chance that droppings may be missed. It is typically difficult to determine in advance whether an observer effect of missed arrivals will be relevant or not. We therefore recommend collecting dropping intervals on single individuals, such that observer bias can be tested for using the presented methodology.

We note that in the design of field studies, it remains important to aim for observational conditions where the probability of missing events of interest p is as low as possible. When p gets large in combination with a standard deviation σ approaching μ in magnitude in equation (3), the interval pdf gets a long tail. In this limit, a model that includes a parameter p will no longer find support in the data over a simpler gamma or exponential model without a parameter p, and the methodology breaks down.

The described interval analysis can be applied beyond the specific case of (geese) defecation to any kind of event history data, especially when inferring the mean and variation in event rates is of interest. Other applications may be observations of diving intervals in birds or mammals (Nolet et al., 1993; Wilson et al., 1995), recording vigilance bouts or scanning frequency in socially foraging animals (Hirschler et al., 2016), or determining prey capture rates in situations where prey captures are occasionally hidden from observers (cf. introduction for more examples). Missed detections also frequently occur in sensor data for which the sampling interval is limited (Wilson et al., 1995), which is the case in many bio‐logger data and automated behavioral classifications based on accelerometers (Shamoun‐Baranes et al., 2012). In these cases, the missed event probability p may be known a priori from the sampling duration in the device settings and may be included as a fixed instead of a free parameter.

Being aware of the problem of missed detections is important in any study that relies on observational data to study behaviors or events that are of short duration and occur at low frequencies. Failure to detect missed detections can both lead to obscuring important variation in the behavior or process under study, or to spurious differences between habitats, groups of animals or social contexts that are in fact due to differences in observation circumstances.

### 4.1 Supporting software

Accompanying this study, we developed the R‐package “intRvals,” available at CRAN (cran.r‐project.org) or Github (github.com/adokter/intRvals), which contains all the tools to perform the analyses presented in this article.
